# Physical exercise-mediated neuroprotective mechanisms in Parkinson's disease, Alzheimer's disease, and epilepsy

**DOI:** 10.1590/1414-431X2024e14094

**Published:** 2024-11-25

**Authors:** R.A. Pinho, A.P. Muller, L.F. Marqueze, Z. Radak, R.M. Arida

**Affiliations:** 1 Laboratório de Bioquímica do Exercício em Saúde, Programa de Pós-Graduação em Ciências da Saúde, Escola de Medicina e Ciências da Vida, Pontifícia Universidade Católica do Paraná, Curitiba, PR, Brasil; 2 Rede Nacional de Neurociência e Atividade Física, Brasil; 3 Departamento de Bioquímica, Universidade Federal de Santa Catarina, Florianópolis, SC, Brasil; 4 Research Institute of Sport Science, Hungarian University of Sport Science, Budapest, Hungary; 5 Departamento de Fisiologia, Universidade Federal de São Paulo, Botucatu, SP, Brasil

**Keywords:** Physical exercise, Neuroprotection, Parkinson's disease, Alzheimer's disease, Glioblastoma, Epilepsy

## Abstract

Research suggests that physical exercise is associated with prevention and management of chronic diseases. The influence of physical exercise on brain function and metabolism and the mechanisms involved are well documented in the literature. This review provides a comprehensive overview of the potential implications of physical exercise and the molecular benefits of exercise in Parkinson's disease, Alzheimer's disease, and epilepsy. Here, we present an overview of the effects of exercise on various aspects of metabolism and brain function. To this end, we conducted an extensive literature search of the PubMed, Web of Science, and Google Scholar databases to identify articles published in the past two decades. This review delves into key aspects including the modulation of neuroinflammation, neurotrophic factors, and synaptic plasticity. Moreover, we explored the potential role of exercise in advancing therapeutic strategies for these chronic diseases. In conclusion, the review highlights the importance of regular physical exercise as a complementary non-pharmacological treatment for individuals with neurological disorders such as Alzheimer's, Parkinson's disease, and epilepsy.

## Introduction

The impact of physical exercise on an individual's overall health and well-being is well established, and regular physical exercise is necessary to prevent and treat numerous chronic diseases. Researchers have gained a substantial understanding of the cellular mechanisms of neurological diseases and revealed that both acute and chronic physical exercise can change brain function through several molecular mechanisms that regulate cellular processes through specific signaling pathways that affect and are affected by the redox and metabolic states of the cell (1). Recent studies clarified how physical exercise modulates neuroinflammatory processes (2, 3), promotes the production of neurotrophic factors (4), increases synaptic plasticity (5, 6), and controls redox balance.

The neuroprotective effects of exercise are evident in several neurological diseases, such as Parkinson's disease (PD), Alzheimer's disease (AD), and epilepsy, highlighting how physical exercise influences cellular neuromechanisms. Despite their different pathophysiology, these neurological disorders exhibit common molecular and redox responses to physical exercise. The impact of physical exercise on brain function and disease is influenced by several factors, including intensity, type, frequency, and duration. For instance, in healthy young adults, a 12-week moderate-intensity continuous training intervention improved cerebral blood flow and executive function more effectively than high-intensity interval training (7). These physical exercise parameters combined with individual biological differences determine how exercise interacts with different brain mechanisms (8). Considering the beneficial impact of exercise on brain health and in various neurological conditions, our primary aim was to provide a comprehensive overview of the potential implications of physical exercise on molecular benefits in Parkinson's disease, Alzheimer's disease, and epilepsy.

We conducted a literature search of the PubMed, Web of Science, and Google Scholar databases to identify manuscripts published within the past two decades. The search strategy involved a combination of keywords and Medical Subject Heading terms. The keywords used included “physical exercise”, “brain metabolism”, “Parkinson's disease”, “Alzheimer's disease”, “epilepsy”, “neurodegenerative diseases”, “neurological disorders”, and “chronic diseases”. Boolean operators (AND and OR) were utilized to refine the search results and ensure a comprehensive collection of relevant studies. We included peer-reviewed articles published in English within the past 25 years (except in some specific cases in which no up-to-date literature was available) that were studies focusing on the impact of physical exercise on brain metabolism and function, research related to specific chronic diseases, and reviews. Articles that did not directly address the connection between physical exercise and brain function, that were not published in English, or were conference abstracts, editorial notes, and commentaries were excluded.

## Parkinson's disease

### Pathophysiological context

PD is a progressive neurodegenerative disease characterized by motor symptoms, such as tremors, rigidity, and bradykinesia, and non-motor symptoms, such as sleep disturbances and cognitive impairment (9). Characteristics of PD include the destruction of dopaminergic neurons in the substantia nigra (the basic nucleus responsible for producing dopamine in the brain) and consequent cytoplasmic inclusions of α-synuclein (α-syn) misfolded aggregates forming Lewy bodies (10). Aggregation and propagation of α-syn in the central nervous system and peripheral tissues play essential roles in the pathogenesis of PD (10).

Pathological mechanisms are strongly associated with several molecular processes including genetic variants, neuroinflammation, and mitochondrial dysfunction. Inflammatory changes are related to the activation of microglia and immune cells in the central nervous system and the release of pro-inflammatory cytokines, chemokines, and other immune mediators leading to the degeneration of dopaminergic neurons (11). Genetic variants associated with PD vary in frequency and risk factors. Several rare variants in single genes, including *SNCA*, which encodes α-syn implicated in Lewy body formation, Parkinson disease protein 7 (*PARK7*), which encodes the protein deglycase DJ-1 involved in oxidative stress protection, and *PRKN*, which encodes parkin, an E3 ubiquitin ligase crucial for maintaining mitochondrial quality control. These and other genetic variants form crucial links between metabolism, inflammation, and mitochondrial dysfunction, all of which contribute to the development of PD (12).

The complex crosstalk between inflammatory processes and mitochondrial dysfunction induces increased oxidative stress associated with PD pathogenesis (13). Immune cell activation within the brain initiates a biochemical cascade that triggers enzymes, such as nicotinamide adenine dinucleotide phosphate oxidase, to produce reactive oxygen species (ROS). This has been extensively documented by the cytokine-induced inflammatory state directly affecting the expression and functionality of critical antioxidant enzymes, which compromises the brain redox balance and exacerbates oxidative stress in PD (14, 15). Mitochondrial dysfunction is characterized by the inefficient management of electron flow across the electron transport chain complexes I-V. This inefficiency promotes electron leakage, notably in complexes I and III, thereby forming superoxide radicals, which are precursors of potent oxidants. The dysfunction of complex I (nicotinamide adenine dinucleotide; ubiquinone oxidoreductase) has emerged as a factor in the pathophysiology of PD. Mutations in genes encoding complex I subunits, as well as environmental toxins such as rotenone, have been implicated in impairing its function, leading to mitochondrial dysfunction and subsequent oxidative stress in PD (16). Moreover, reduced complex I activity has been observed in the substantia nigra, the brain region primarily affected in PD, contributing to the selective vulnerability of dopaminergic neurons (14).

### Physical exercise-mediated molecular regulation in PD

**Figure 1:**
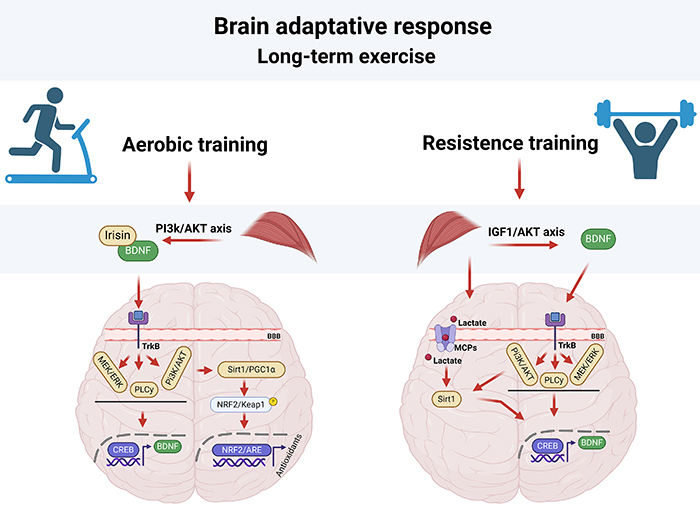
Molecular mechanisms induced by long-term exercise in the brain’s adaptive responses. This schematic represents the differential pathways activated in the brain as a response to aerobic and resistance training. The aerobic exercise triggers the release of muscle-derived exerkines, which in turn enhance the expression of brain-derived neurotrophic factor (BDNF) via the PI3K/AKT signaling pathway. This cascade promotes neural survival and synaptic plasticity through multiple intermediates mediated by TrkB receptor. Additionally, the Sirt1/PGC1α pathway regulates antioxidant gene transcription via NRF2/ARE to combat oxidative stress. In response to resistance training, two parallel yet distinct pathways are initiated, involving IGF1/AKT signaling and lactate-activated sirtuins, which converge on BDNF synthesis and activate epigenetic regulators involved in DNA repair, thereby enhancing neuronal resilience.

Pre-clinical, clinical, and epidemiological studies have revealed the potential effects of non-pharmacological therapies for PD, as physical exercise mitigates changes in neuroinflammation, oxidative stress, and mitochondrial dysfunction (17-21) and improves motor and non-motor symptoms (22-24). Integrating non-pharmacological therapies with conventional treatments can improve long-term therapeutic outcomes. Although influenced by the intensity and duration of exercise, an acute response to physical exercise increases blood flow and neurotrophic factors (25, 26), whereas adaptive responses include the upregulation of antioxidants and repair enzymes and redox regulation by different neurotrophic, synaptogenic, angiogenic, and neurogenic processes (27) (Figure 1).

The cellular mechanisms underlying the benefits of physical exercise on motor and non-motor symptoms in PD are not yet fully understood. However, evidence suggests that exercise-sensitive molecular mediators and neuroplasticity processes are associated with these symptoms. For example, studies have revealed that the upregulation of brain-derived neurotrophic factor (BDNF) is among the main molecular mechanisms induced by physical exercise in patients with PD, which may improve motor and non-motor symptoms (3,4,19,28-30). BDNF belongs to a family of neurotrophic factors that bind to and activate tropomyosin receptor kinase B (TrkB), which regulates several small G proteins, including Ras, mitogen-activated protein kinase (MAPK), PI3-kinase, and phospholipase-C-γ (PLC-γ) pathways. As a result of the interaction of BDNF with its receptor, other signaling pathways (Ras/MAPK/extracellular signal-related kinase and IRS-1/phophoinositide 3-kinase/AKT PLC-γ/diacylglycerol/inositol 1,4,5-triphosphate pathways) are activated, which in turn activate one or both transcription factors, including the cyclic adenosine monophosphate (cAMP) response element-binding protein (CREB), which regulates the expression of genes encoding proteins involved in the survival, development, and function of neurons during synaptogenesis, synaptic plasticity, and cognitive functions (4, 29).

Different types of physical exercise increase the BDNF levels in the blood (30-32) and the brain (18, 33). BDNF levels in the peripheral and specific brain regions promote different responses, each contributing uniquely to the neuroprotective effects of physical exercise. For example, serum BDNF levels can reflect systemic changes and overall neurotrophic support (29, 31). BDNF levels in brain regions are directly related to local neuronal health, synaptic plasticity, and cognitive function, providing targeted neuroprotection and promoting neuronal survival (33, 34).

Both aerobic and strength training exercises offer significant benefits in mitigating the motor and non-motor symptoms of PD by leveraging the mechanisms of neuroprotection and neuroplasticity associated with increased BDNF expression (34). Different exercise-dependent molecules or signaling pathways have been suggested to upregulate BDNF expression. Resistance exercise activates important signaling pathways within neurons, including the insulin-like growth factor 1/CREB, MAPK/extracellular signal-regulated kinase and phosphoinositide 3-kinase/Akt/mammalian target of rapamycin pathways, which promote *BDNF* gene expression and protein synthesis (35). Increased lactate levels due to exercise enhance *BDNF* signaling in the hippocampus and promote learning and memory (36). Under oxidative stress conditions, aerobic physical exercise contributes to redox balance by increasing the expression of nuclear factor erythroid 2-related factor 2 (Nrf2), which upregulates antioxidant genes (37) and exerts anti-ferroptotic effects on PD (38). Nrf2 acts directly on brain redox regulation and promotes transcriptional activity, increasing BDNF expression and crosstalk between antioxidant signaling pathways and neurotrophic factors. This hypothesis is supported by findings of previous studies. Increased BDNF levels have been observed in cancer-associated fibroblasts with activated TrkB-Nrf2 signaling (39). Yao et al. (40) showed that the activation of Nrf2 by sulforaphane or *Nrf2* gene transfection increased the BDNF mRNA expression and that Nrf2 binds to the BDNF exon I promoter, indicating its function as a transcription factor for BDNF.

In summary, the mechanisms regulated by physical exercise in patients with PD modulate neurotrophic factors and oxidative stress, thereby contributing to synaptic plasticity, inflammatory control, and mitochondrial function. Exercise characteristics, such as type, intensity, frequency, and duration, should be considered to promote long-term neuroprotective responses.

## Alzheimer's disease

### Pathophysiological context

AD is the most prevalent neurodegenerative disorder worldwide. The accumulation of β-amyloid plaques and neurofibrillary tangles is central to AD pathology, as it disrupts synaptic function, triggering neuroinflammatory responses (41). Moreover, the etiology of AD is complex and involves a combination of genetic and environmental factors that affect brain metabolism (42).

The brain’s glucose metabolism is impaired in AD patients (36). Brain metabolism requires considerable energy and high O_2_ consumption by mitochondrial oxidative phosphorylation, which can trigger ROS production. Brain cells have a low antioxidant capacity, and mitochondrial dysfunction in complex IV is associated with AD (43). AD has a pre-clinical phase that can last for years. During this time, changes in the brain occur even though no symptoms have yet appeared (41). Although some drugs have shown promising results, no effective treatments have been identified to date (44). Environmental factors such as physical activity and a healthy diet play crucial roles in the prevention and treatment of AD (45).

Mitochondrial dysfunction, a critical factor in the pathophysiology of AD, is characterized by impaired oxidative phosphorylation and increased ROS levels (46). Moreover, β-amyloid disrupts mitochondrial dynamics, increases Fis1, the protein involved in fission, and decreases the expression of fusion proteins such as DLP1, OPA1, Mfn1, and Mfn2. ROS can damage membranes, proteins, and DNA of brain cells, and oxidative stress markers have been identified in AD (47). Furthermore, mitochondrial dysfunction plays a role in diminishing adenosine triphosphate (ATP) production, disrupting Ca^2+^ homeostasis and causing changes in mitochondrial dynamics and mitophagy during the early stages of AD (46). While AD is progressive, aerobic exercise interventions may help slow global cognitive decline and enhance specific memory functions (45).

### Physical exercise-related molecular modulations in AD

**Figure 2:**
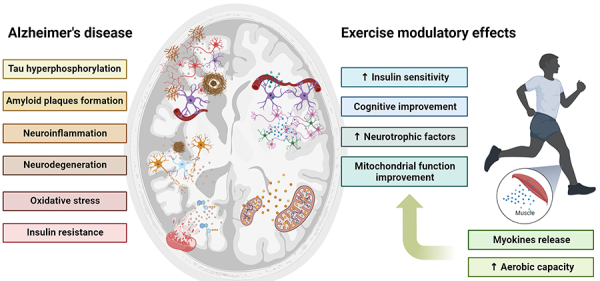
Protective effects of exercise on Alzheimer's disease pathology. The release of myokines from skeletal muscle during physical activity and increased aerobic capacity benefits the central nervous system. The left side of the figure outlines the pathological features characteristic of Alzheimer's disease, including hyperphosphorylation, amyloid plaque formation, neuroinflammation, neurodegeneration, oxidative stress, and insulin resistance. The right side illustrates how regular exercise exerts beneficial modulatory effects on Alzheimer's disease, including insulin sensitivity, cognitive enhancement, and elevation of neurotrophic factors that promote neuronal health and survival. Exercise also induces improvement in mitochondrial function, essential for energy homeostasis and neuronal function.

Regular exercise has been shown to promote brain health, serving as a preventive measure against AD (30, 48). Physical activity was inversely associated with the risk of dementia in 163,000 participants without dementia (49). Physical exercise-mediated metabolic and molecular alterations are shown in Figure 2.

Mitochondrial DNA oxidation is observed in the initial phases of AD, resulting in impaired glucose metabolism. However, physical exercise enhances the DNA repair system by increasing BDNF, CREB, and APE1 signaling (50). Improvement in the antioxidant activity of enzymes is a remarkable effect of exercise on the brain, which protects against damage caused by excessive O_2_ consumption (51). Exercise improved antioxidant enzyme levels (50) in transgenic female mice with AD. Physical activity was shown to increase mitochondrial activity and brain energy, both of which are essential for learning and memory (52). Physical training improves cognitive domains and decreases oxidative marker levels in patients with AD (53).

Exercise improves brain metabolism, blood flow, and mitochondrial function by stimulating peroxisome proliferator-activated receptor-gamma coactivator-1α and Nrf-2. Furthermore, exercise increases brain antioxidant levels by improving the activities of antioxidant enzymes such as superoxide dismutase, catalase, and glutathione peroxidase (54). These findings highlight the role of exercise in the prevention and treatment of AD (55). Improvements in aerobic capacity are associated with slower progressive cognitive impairment observed in AD (55), enhanced hippocampal activity, and compensation for age-related anterior hippocampal volume loss. Resistance exercise increases prefrontal cortex activity (56), induces an exercise-dependent increase in neurochemical markers of brain neuronal density, and enhances neurotransmission as observed using proton magnetic resonance spectroscopy (57).

Physical exercise stimulates muscle cells to produce bioactive molecules known as myokines. More than 600 myokines have been identified and described to date. Irisin is a physical exercise-induced myokine that is released upon cleavage of the membrane-bound precursor protein fibronectin type III domain-containing protein 5 (FNDC5), which potentially modulates AD (48). However, whether exercise increases irisin levels remains controversial. Neither acute nor chronic endurance or resistance exercise led to increased FNDC5 expression or circulating concentrations of irisin, whereas 20 days of high-intensity interval training led to acute increases in the muscle expression of FNDC5 in healthy men (27). Another myokine that induces brain modulation is cathepsin B, which is upregulated by exercise in animal models as well as in monkeys (58). Irisin and cathepsin B improve the function of BDNF, a neurotrophic factor that increases hippocampal neurogenesis, synaptic plasticity, spinal dendritic cells, neurotransmitter receptors, and synaptic proteins, thereby improving learning and memory (59). In an animal model of AD, neurotrophic factors are decreased and associated with cognitive deficits (60), whereas exercise is a known neurotrophic inducer (61).

Neuroinflammation and microglial hyperactivation change brain cell structure, metabolism, and function and are among the most important factors in AD development (62). The microglial activation increases cytokine synthesis and actions, such as interleukin (IL)-1β, IL-6, and tumor necrosis factor-α, which impair learning and memory (63). Physical exercise decreases inflammatory processes and improves anti-inflammatory pathways, even in the brain, and it has been designated as an anti-AD factor (64). Cytokines released into the bloodstream by muscle fibers are expected to regulate physical exercise-induced pro- and anti-inflammatory states. Resistance exercise increases anti-inflammatory cytokine IL-10 levels in the hippocampus of healthy old male rats and in the frontal cortex of male AD mice (30). Patients with AD showed increased inflammatory biomarkers, and after exercise intervention, these biomarkers were modulated, including increased levels of IL-6, a myokine that has an anti-inflammatory effect on the brain (65). IL-6 upregulates the expression of the anti-inflammatory cytokine IL-10 and increases the levels of IL-1 and IL-1 receptor antagonists. Moreover, IL-6 can downregulate the expression of pro-inflammatory factors including tumor necrosis factor-α or IL-1β (66).

Insulin is an important regulator of metabolism and exerts anabolic effects on the brain. Insulin is currently recognized as a neurotrophic factor whose impairment in the brain causes AD. Brain insulin resistance is a key factor in AD, which is treated as type III diabetes (67). Intranasal insulin administration is a potential treatment option for AD (68). Insulin signaling improves synaptogenesis, decreases tau hyperphosphorylation, increases β-amyloid clearance, and promotes cell survival (69). Exercise increases insulin sensitivity in the hippocampus, a brain structure that is more severely affected by AD (70). Moreover, lifestyle changes, including increased physical activity, increased intranasal insulin signaling (71). Furthermore, exercise has the potential to modulate insulin transport across the blood-brain barrier and enhance insulin signaling in various brain regions, as demonstrated in young mice (72).

## Epilepsy

### Pathophysiological context

Epilepsy is among the most common neurological disorders that can occur at any age, affecting approximately 1% of the global population (73). It is characterized by spontaneous recurrent epileptic seizures, which are the hallmarks of epilepsy and can result in several neurobiological, cognitive, and psychosocial alterations (74). According to the International League Against Epilepsy (ILAE), epilepsy is diagnosed when a patient has two unprovoked seizures occurring more than 24 h apart, when someone has a single unprovoked seizure (if the recurrence risk is high), or when a diagnosis of epilepsy syndrome is confirmed (74). The ILAE Task Force established six etiological categories to classify the causes of epilepsy: genetic, structural, metabolic, infectious, immune, and unknown (75). Mesial-temporal lobe epilepsy is the most common form in humans. Its pathophysiological substrate is hippocampal sclerosis, the most common epileptogenic lesion in patients with epilepsy. Common pathological findings of hippocampal sclerosis include atrophy and astrogliosis of the hippocampus, amygdala, parahippocampal gyrus, and entorhinal cortex, characterized by a decreased number of pyramidal and hilar neurons and loss in other extratemporal regions (76).

Seizures and epilepsy result from diverse pathological processes that disrupt the balance between excitation and inhibition. They disrupt extracellular ion homeostasis, alter energy metabolism, modify receptor function, and affect transmitter uptake. In this context, increased attention has been directed toward the roles of neuroinflammation and oxidative stress in the pathophysiology of epilepsy. Clinical and experimental evidence strongly supports the hypothesis that inflammatory processes within the brain represent common and crucial mechanisms in the pathophysiology of seizures and epilepsy (77). Experimental studies have demonstrated that seizure activity can trigger brain inflammation, and recurrent seizures contribute to chronic inflammation (78). In response to injury, inflammation promotes the utilization of ROS to combat pathogens and facilitates rapid local signaling. ROS are important secondary messengers that modulate neuroinflammation at various stages through redox-sensitive mechanisms. ROS overproduction or redox dysregulation under oxidative stress can lead to cellular damage, triggering danger signals that cause neuroinflammation (79). Mitochondrial dysfunction is an important pathological characteristic that exacerbates the inflammatory process associated with epilepsy. Approximately 40% of patients with epilepsy also have mitochondrial diseases (80). Therefore, interventions that target neuroinflammation, including interventions that affect mitochondria, can potentially achieve better clinical outcomes in epilepsy.

### Physical exercise-mediated redox and metabolic regulation in epilepsy

Although most therapeutic procedures to control seizures are pharmacological, non-pharmacological approaches, including complementary medicine, are also often used (81). In this scenario, physical exercise is considered a potential complementary therapy for epilepsy (82), and an extensive number of clinical and non-clinical studies have contributed to the knowledge of the beneficial effects of exercise on epilepsy (83). In the past, patients with epilepsy were often advised not to participate in physical and sports activities, mostly because of fear, overprotection, and ignorance about the benefits and risks associated with such activities. However, there is a strong consensus that people living with epilepsy should increase their physical activity (84).

Investigations primarily focusing on the effects of physical exercise programs on seizure frequency have reported improvements in seizure control (85, 86) or no increase in seizure frequency after physical exercise interventions (87). Studies showing no increase in seizure frequency after a physical exercise reported that seizures occur more often during rest compared with the training period (87). Therefore, most patients were seizure-free at baseline and during training (88). In addition, studies analyzing whether intensive exercise could interfere with seizure susceptibility did not report seizures being induced incrementally by exercise to exhaustion on ergometric tests or after physical exertion (89, 90). The psychological effects of physical exercise on individuals with epilepsy, including depression, mood, and quality of life, have also been documented (88, 91).

**Figure 3:**
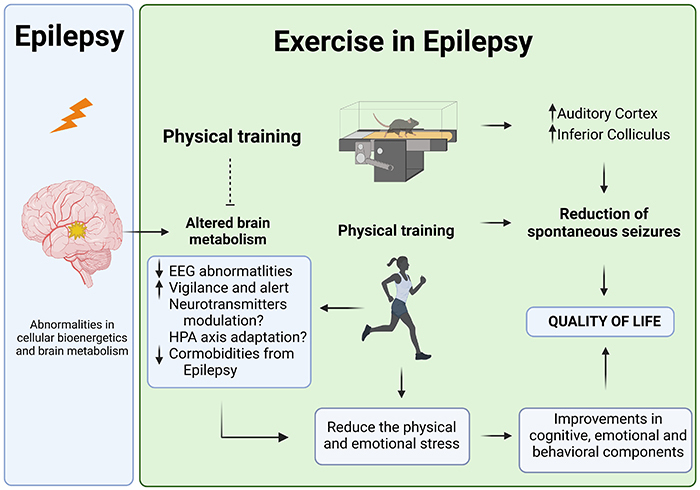
Summary of proposed mechanism by which exercise improves the altered metabolism in the epileptic brain. Modulation of hypothalamic-pituitary-adrenal axis (HPA), neurotransmission/neuromodulation, and neuroinflammation systems are possible mechanisms. Although not yet investigated in humans, an increase in cerebral metabolism is observed in the inferior colliculus and auditory cortex of trained rats with epilepsy compared to non-trained rats with epilepsy. These mechanisms have also been suggested to reduce or suppress spontaneous seizures and improve the quality of life of people with epilepsy.

The proposed mechanisms by which regular physical exercise can reduce the susceptibility to seizures have been highlighted in several animal models of seizures and epilepsy (Figure 3). Among these, the modulation of neurotransmitters, neurotrophic factors, and brain metabolism are key factors that may interfere with seizure susceptibility. Other mechanisms that have been poorly investigated in epileptic conditions include the hypothalamic-pituitary-adrenocortical axis, neurosteroid and steroid hormones, and the opioid, inflammatory, and adenosinergic systems (83, 92).

The brain is a vital organ with a high metabolic demand, and it is challenged to raise its metabolic rate in response to physical exercise. Recurrent or prolonged seizures induce abnormalities in cellular bioenergetics and brain metabolism (93) with extensive disturbances in homeostatic mechanisms. Increased neuronal excitability is supported by increased brain metabolism (94), and several lines of evidence indicate that metabolic changes are the cause and/or consequence of seizure activity (95). Therefore, brain metabolism during seizures and the interictal period (the time between seizures) provides a valuable indication of the brain structures responsible for the generation, propagation, and control of epileptic activity. Although acute seizures increase cerebral blood flow and local cerebral glucose levels, resulting in increased lactate levels and a reduction in phosphocreatine and ATP levels (96) during the interictal period, hypometabolism in several brain structures has been observed in both human and animal models of epilepsy (97, 98).

During physical exercise, an increase in the requirements for oxygen and substrates is observed in the brain (99, 100). Increased cerebral neural activity and metabolism (101) are followed by transient enhancement of cerebral blood flow during and/or after exercise (102, 103). Although several studies have shown altered cerebral metabolism during physical exercise, information on the effects of physical exercise on cerebral metabolism in patients with epilepsy is scarce. Using local cerebral metabolic rates for glucose (LCMRglu) measured by the quantitative [^14^C]2-deoxyglucose method, Arida et al. (104) investigated whether a physical exercise program could modify functional activity in brain areas using the pilocarpine animal model of temporal lobe epilepsy. Considering that seizures usually occur at rest and not during exercise, LCMRglu was measured during the interictal period of epileptic seizures. Trained rats with epilepsy showed an increase in interictal LCMRglu in the inferior colliculus and auditory cortex compared with control rats with epilepsy. In addition, the aerobic exercise program reversed the low metabolic rates in several structures in animals with epilepsy to prevent seizures. Similarly, higher local cerebral glucose utilization during exercise has been demonstrated in the auditory and visual cortices, the areas involved in attention, vigilance, and alertness, suggesting that these alterations are not directly related to exercise but rather to higher mental alertness during physical activity (99). Considering that a certain level of alertness is necessary during physical or sports activities, increased vigilance and attention during exercise may be involved in seizure reduction (105). However, these changes in cerebral metabolic rate were observed at rest and not measured during physical exercise. Therefore, the increased metabolic rate in these structures may explain the reduction in the number of seizures reported in previous studies of trained animals with epilepsy (106, 107).

Other metabolic mechanisms have not yet been explored. Adenosine, a by-product of energy metabolism and ATP utilization, can act as a seizure inhibitor. During metabolic stress, extracellular adenosine concentrations increase rapidly, activating adenosine receptors (108, 109). Increased extracellular adenosine is accompanied by augmented metabolic activity, and seizures induce an increase in extracellular adenosine. During intensive physical exercise, the brain prefers lactate to glucose as its primary energy substrate, thereby increasing ATP production (110). Intensive exercise causes metabolic acidosis by increasing serum lactate content. Acidosis serves as a protective mechanism by increasing the seizure threshold (89). Acidosis increases gamma-aminobutyric acid levels, whereas alkalosis decreases them. Indeed, an initial investigation suggested that decreased epileptogenic EEG activity during physical effort might be related to increased gamma-aminobutyric acid concentrations due to metabolic acidosis (89).

### Mitochondrial dysfunction in epilepsy

Focal, generalized, and myoclonic seizures and epileptic spasms are the most common forms of seizures associated with mitochondrial diseases (111). Studies have indicated that seizures in patients with mitochondrial disease may be induced by excessive amounts of ROS resulting from abnormal mitochondrial function (112, 113). Seizures occur due to an energy deficit, specifically ATP, resulting from impaired oxidative phosphorylation, which is central to mitochondrial diseases. ATP molecules are crucial for the function of sodium-potassium ATPase, which ensures normal polarization of the neural membrane. Impairment of the membrane potential induces neuronal hyperexcitability, resulting in seizures (114). Decreased ATP production produced by malfunctioning brain cell mitochondria leads to disrupted metabolism and seizures (115). Thus, the association between epilepsy and mitochondrial diseases can be characterized by disrupted energy metabolism and abnormal neuronal activity.

Although the literature has demonstrated that the relationship between mitochondrial dysfunction and ROS generation contributes to the mechanisms that induce seizures, there is limited information on the mechanisms by which physical exercise affects mitochondrial dysfunction in epilepsy. It is well known that regular exercise induces increased endurance capacity resulting from mitochondrial biogenesis, reduced oxidant production, and enhanced antioxidant defense (116, 117). To our knowledge, only one study in this context has investigated the anticonvulsant effect of a physical exercise program on seizures induced by pentylenetetrazole using an animal model of traumatic brain injury. In another study (118), treadmill exercise reduced redox status alterations, characterized by lipid peroxidation and protein carbonyl increase, and inhibited superoxide dismutase and Na+/K+ ATPase activities after traumatic brain injury. Although further investigations are required to better understand the effects of exercise on mitochondrial dysfunction in epilepsy, exercise may be an important approach to inducing mitochondrial adaptive responses to control seizure activity.

Considering that physical exercise modulates hypothalamic-pituitary-adrenal activity and the synthesis and release of several neurotransmitters such as serotonin, noradrenaline, and dopamine as well as upregulates neurotrophic factors, exercise under epileptic conditions may reduce stress and decrease seizure susceptibility. Some mechanisms responsible for the beneficial effects of physical exercise in chronic epilepsy appear to be related to the regulation of brain activity, such as reducing seizure susceptibility, anxiety, and depression as well as improving socialization, increasing self-esteem, and improving the quality of life. Thus, physical exercise is a potential candidate for integration with conventional therapies for the treatment of epilepsy (84).

## Perspectives

Studies of the effects of physical exercise on neurological diseases are a rapidly evolving field with promising future perspectives. Further studies are required to investigate the relationship between physical exercise and chronic neurodegenerative diseases. However, it is necessary to understand the different types, intensities, durations, and frequencies that exert neuroprotective effects in PD, AD, and epilepsy. The heterogeneity of studies on PD, AD, and epilepsy complicates direct comparisons among them. Participant individuality, including differences in age, disease severity, and fitness level, hinders the generalizability of our findings. The standardization of methodologies and adoption of uniform criteria for assessing disease progression and exercise interventions can improve the comparability of findings across studies.

Another important future direction is the integration of exercise interventions with other therapeutic approaches such as pharmacological treatments or noninvasive brain stimulation techniques. Combining exercise with these approaches may have synergistic effects and improve the outcomes of patients with neurological diseases. Moreover, the use of technologies and digital health interventions, such as smartphone apps or virtual reality platforms, can facilitate the delivery of exercise interventions and promote patient adherence. Future studies should explore the effectiveness of these technologies in promoting exercise and improving the outcomes of patients with neurological diseases. Overall, future studies involving physical exercise in neurological diseases are promising, and further research in this field has the potential to improve the quality of life of millions of individuals with these conditions.

Although animal studies provide important data, the translation of these findings into effective clinical interventions requires well-designed and extensive trials. Addressing these limitations will improve our understanding of the relationship between exercise and neurodegenerative diseases and pave the way for evidence-based recommendations. In conclusion, understanding the mechanisms by which physical exercise can positively influence the treatment of the aforementioned neurological diseases is crucial for establishing appropriate guidelines that can improve public health.
